# Supplementary material for: Outcome after Prenatal Diagnosis of Trisomy 13, 18, and 21 in Fetuses with Congenital Heart Disease
Source: Life (Basel). 2022 Aug 12;12(8):1223. doi: 10.3390/life12081223 (PMC9410270; doi:10.3390/life12081223)
Supplement: Supplementary file 1 [file life-12-01223-s001.zip › life-1844248-supplementary.pdf]

Supplementary Table S1: Peri- and postnatal details of mortality in children with congenital heart disease (CHD) and trisomy 13, 18, and 21

Trisomy/ Case	GA at diagnosis	IUGR	CHD	Extracardiac anomalies	GA at birth	Mode of delivery	Birthweight (percentile)	Sex	Outcome
13/1	12+5	no	TAC	0	40+1	Vaginal	2930 (5)	m	Death at 40 hours
13/2	22+0	yes	HRHS	Hexadactyly	36+0	Cesarean	1663 (<3)	f	Death at 6 hours
13/3	24+1	yes	DORV	Microcephaly, cerebellar agenesis, MMC, micrognathy, enlarged kindneys, micropenis	38+3	Vaginal	1710 (<3)	m	Death at 15 minutes
13/4	27+3	yes	DORV, RAA, PA	Polydactyly, micrognathy, SUA	36+1	Cesarean *	2240 (7)	m	Death at 3 ½ hours
13/5	30+5	yes	VSD	Holoprosencephaly, CLP, hypotelorism, arrhiny, enlarged kidneys, omphalocele	36+2	Vaginal *	1640 (<3)	f	Death at 7 hours
18/6	21+1	yes	DORV, CoA	Strawberry-shaped skull, SUA	41+3	Vaginal	2504 (<3)	m	Death sub partu
18/7	22+6	yes	DORV	Strawberry-shaped skull, micrognathy, SUA	36+1	Vaginal	1490 (<3)	m	Alive after outpatient delivery, palliative care, time of death unknown
18/8	23+5	yes	VSD	Clubfeet, clenched hands	28+5	Cesarean **	810 (8)	m	Death at 4 days
18/9	30+1	yes	DORV, AVSD	Strawberry-shaped skull, hypotelorism, micrognathy, clenched hands, enlarged bladder	34+0	Vaginal *	990 (<3)	f	Death after 18 ½ hours
18/10	35+0	yes	VSD	Dandy-Walker malformation, hydramnios	40+0	Vaginal	2250 (<3)	f	Death after 5 ½ hours
21/11	20+0	no	VSD, ASD II	Craniofacial dysmorphism, abnormal cerebral develop-ment, clenched hands	38+5	Cesarean *, ***	3368 (44)	m	Death at 10 months, chondrodysplasia punctata
21/12	21+6	no	DORV, AVSD	Ventriculomegaly	27+3	Cesarean #	730 (9)	m	Death at 18 days, sepsis
21/13	26+5	yes	VSD, ASD, CoA	0	32+4	Cesarean #	1300 (4)	m	Death at 22 days, hydrops, sepsis
21/14	29+1	no	AVSD	Esophageal atresia	34+2	Cesarean #	1830 (<3)	m	Death at 1 month, sepsis
21/15	33+4	no	AVSD, TOF	0	39+1	Cesarean ***	4230 (95)	m	Death at 3 hours, hydrops, sepsis

GA, gestational age; IUGR, intrauterine growth restriction; TAC, Truncus arteriosus communis; HRHS, hypoplastic right heart syndrome; DORV, double-outlet right ventricle; RAA, right-sided aortic arch; PA, pulmonary atresia; VSD, ventricular septal defect; CoA, coarctation of the aorta; ASD, atrial septal defect; TOF, tetralogy of Fallot; MMC, meningomyelocele; SUA, single umbilical artery; CLP, cleft lip and palate.

*maternal pre-eclampsia

**premature rupture of membranes

***maternal gestational diabetes requiring insulin

highly abnormal fetal Doppler: zero/reversed flow in the umbilical artery.
